# Economic evaluation on meningococcal vaccination strategies among children under nine years of age in Zhejiang province, China

**DOI:** 10.1371/journal.pone.0310274

**Published:** 2024-09-09

**Authors:** Jianyong Shen, Chai Ji, Xiaofu Luo, Yu Hu

**Affiliations:** 1 Institute of Immunization and Prevention, Zhejiang Provincial Center for Disease Control and Prevention, Hangzhou, China; 2 Institute of Immunization and Prevention, Huzhou Municipal Center for Disease Control and Prevention, Huzhou, China; 3 Department of Children Healthcare, Children’s Hospital Affiliated to Zhejiang University School of Medicine, Hangzhou, China; Public Health England, UNITED KINGDOM OF GREAT BRITAIN AND NORTHERN IRELAND

## Abstract

Meningococcal vaccination in Chinese national immunization program (NIP) includes polysaccharide vaccine against Neisseria meningitidis serogroup A (MPV-A) and polysaccharide vaccine against Neisseria meningitidis serogroup A and C(MPV-AC). This study aimed to assess the cost-effectiveness of an alternative strategy using polysaccharide conjugate vaccine against Neisseria meningitidis serogroup A,C,W,Y(MCV-ACWY) and polysaccharide vaccine against Neisseria meningitidis serogroup A,C,W,Y(MPV-ACWY). From a societal perspective, we constructed a decision tree-Markov model to simulate the economic and health consequences of meningococcal disease in a 2023 birth cohort with the current meningococcal vaccination strategy and the alternative. Parameters of epidemiology, vaccine efficacy, cost, and utility were extracted from database and previous literatures. The sensitivity analysis was implemented to evaluate the robustness of the model. Compared to the current practice, the alternative strategy could avoid 513 meningococcal disease cases, 53 sequelae and 47 deaths. The ICER was estimated at $16899.81 /QALY, under the threshold of one time of the GDP per capita of Zhejiang province in 2023. The incidence of meningococcemia, the incidence of meningococcal meningitis, the case fatality of meningococcemia, the vaccine efficacy of MCV-ACWY and the price of MCV-ACWY would influence the cost-effectiveness of the meningococcal vaccination strategies. At the threshold, the probability of cost-effectiveness was 14.76% for the current strategy and 55.98% for the alternative strategy, respectively. The current meningococcal vaccination strategy had effectively prevented meningococcal disease at a low cost, but with limited serogroup coverage. Strategy using MCV-ACWY and MPV-ACWY could increase health benefits at a substantial cost at a cost-effective manner.

## 1. Introduction

Meningococcal disease is caused by the Neisseria meningitidis, which is classified as 12 serogroups, with serogroup A (MenA), serogroup B (MenB), serogroup C (MenC), serogroup W (MenW), serogroup X (MenX), serogroup Y (MenY) being the main serogroups associated with current cases [1]. The most common clinical manifestations for meningococcal disease are meningitis and septicemia. The mortality of meningococcal disease is 10% worldwide, even with appropriate antibiotic therapy. The case fatality rate of meningococcemia is up to 40% [2]. Approximately 10–20% of patients who survive meningococcal disease have permanent sequelae, such as hemiplegia, deafness, skin grafting and seizures [2]. Humans are the only natural reservoir of Neisseria meningitidis. Carrier rate is usually highest among adolescents and young adults (4.38%) [3]. Aerosol droplets or direct contact leads to transmission through respiratory secretions and requires close contact [3].

In China, the incidence of meningococcal disease was highest in 1967, with an incidence rate of 403/100,000 and a fatality rate of 5.5% [4]. Through using polysaccharide vaccine against MenA (MPV-A), the incidence of meningococcal disease has remained <1/100,000 since the 1990s, and no pandemic or outbreak has occurred since 2003 [4]. In China, the average cost of meningococcal disease was 73000 RMB, almost equal to 2.5 times of Gross Domestic Product (GDP) per capita in China during 2007–2008 [5]. MenC was the predominant circulating serogroup from 2003 to 2014, accounting for 59.6% of cases [6]. MenB exhibited an upward trend from 2010 to 2020, and the share of meningococcal disease cases due to MenB was 36.15%, followed by MenC (22.97%), MenW (6.08%) and MenA (4.73%) [7]. The incidence of meningococcal disease due to MenA and MenC decreased significantly due to the mass vaccination induced since the inclusion of MPV-A in national immunization program (NIP) in 2003 and polysaccharide vaccine against MenA and MenC (MPV-AC) in 2008 [8]. The coverage rates of MPV-A and MPV-AC have maintained >90% for the last decade. The vaccination coverage of meningococcal vaccines ranged from 88%-89% among adolescents aged 13-17years in USA in 2023 [9] and ranged from 54%-91.4% for adolescents aged 16 and 18 years in Italy in 2022 [10]. The proportions of the meningococcal disease cases aged 0–5 years, 6–10 years, ≥ 18 years were 29.6%, 28.9% and 19.2%, respectively [11]. Children face a significant high risk of infection of Neisseria meningitidis. However, NIP only offer two doses of MPV-A at 6 and 9 months of age and two doses of MPV-AC at 3 and 6 years of age. As a supplement, several voluntary meningococcal vaccines have been licensed for use in children and adolescents, including polysaccharide vaccine against MenA, MenC, MenW, MenY (MPV-ACWY), polysaccharide conjugate vaccine against MenA and MenC (MCV-AC), polysaccharide conjugate vaccine against MenA, MenC, MenW, MenY (MCV-ACWY). These voluntary vaccines are available for eligible children and adolescents at their own expense [4].

The immunogenicity, efficacy, effectiveness, duration of protection and safety of polysaccharide conjugate vaccine are better than polysaccharide vaccine. The strengths of polysaccharide conjugate vaccine include the immunological memory, higher affinity of antibody, effective protection in toddlers under 2 years of age who do not respond to polysaccharide vaccine. In a comparative safety and immunogenicity trial of meningococcal vaccines, all four serogroups showed significantly higher seroconversion rate in the MCV-ACWY group than in the MPV-ACWY group [12]. A review of effectiveness of meningococcal vaccines indicated the effectiveness rate of 65.0–83.7% for polysaccharide vaccine and 66.0–100.0% for polysaccharide conjugate vaccine, concluding polysaccharide conjugate vaccine had the higher effectiveness and long-lasting protection [13]. Since the polyvalent polysaccharide conjugate meningococcal vaccine can offer a more comprehensive and effective protection, global trends of vaccination strategies towards meningococcal disease had shifted to the replacement of monovalent vaccines with polyvalent vaccines and the replacement of polysaccharide vaccines with polysaccharide conjugate vaccines. Many developed countries had included MCP-ACWY into their NIPs. However, Chinese NIP only incorporated polysaccharide meningococcal vaccine that cover limited serogroups, leaving the risk of outbreaks by other serogroups and poor protection in age groups under 2 years of age.

The upgradation of vaccination strategy against meningococcal disease in line with the global trends is necessary and should be put on agenda as soon as possible. Supporting evidence under the local context is important for policy-making on vaccination strategy. The evidence includes but not limited to the health outcomes from an epidemiological perspective and costs from an economic perspective to determine whether the vaccination program is worthwhile to invest. Although the literature on economic evaluation on meningococcal vaccine is abundant in many countries, while the existing reports in China are quite limited. There is no economic evaluation on vaccination strategies by using the voluntary meningococcal vaccine in China.

This study aimed to implemented a health economic evaluation to compare the meningococcal vaccination strategy incorporating MPV-ACWY and MCV-ACWY into NIP with the current NIP strategy in the context of Zhejiang province, China.

## 2. Methods

Compared with the current meningococcal vaccination strategy, the cost-effectiveness analysis of the alternative meningococcal vaccination strategy incorporating MPV-ACWY and MCV-ACWY into NIP for children was performed from a societal perspective.

### 2.1 Vaccination strategy

The meningococcal vaccination strategy in NIP was established as the baseline against the alternative strategy. The NIP vaccines can reach a coverage of 90% among the targeted children in China. In this study, the alternative meningococcal vaccination strategy assumed that the voluntary vaccine would be integrated into NIP to ensure free for target children. The schedule of the alternative meningococcal vaccination strategy included 3 doses of MCV-ACWY at 3,4,5 months of age and 2 doses of MPV-ACWY at 3 and 6 years of age, which was based on the package instructions and common practice.

### 2.2 Model construction

**Fig 1:**
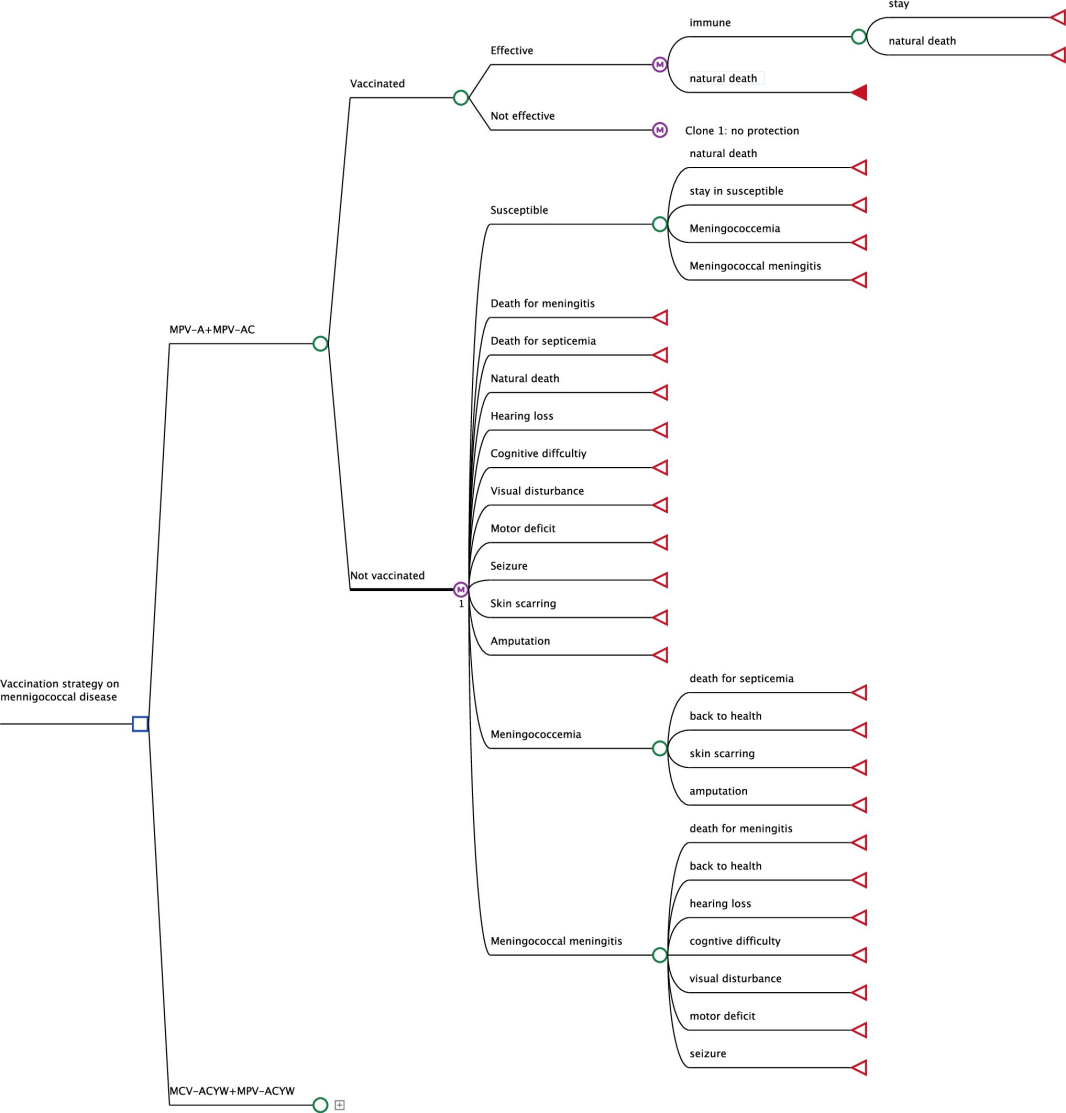
Overview of the decision tree-Markov model.

A decision tree-Markov model was constructed in TreeAge Pro 2019 on a cohort of 385266 newborns in 2023, in Zhejiang province (Fig 1). Simulations were implemented on a cycle of one year, from birth to 9 years of age. The reason for determining the simulation period was based on the vaccination strategy and the duration of protection of 3 years and 5 years for meningococcal polysaccharide vaccine and meningococcal polysaccharide conjugate vaccine, respectively [14, 15]. A life expectancy of 79 years was adopted as the time horizon, accounting lifetime costs and impacts. We assumed that the serogroup replacement of Neisseria meningitidis would not impact the incidence of meningococcal disease as the duration of simulation was relatively short. Herd immunity was not considered, since a high vaccination coverage was set in the model. Almost all children would get vaccinated under a coverage of 90%, leading to very few children in the cohort who need indirect protection by herd immunity [16]. Health outcomes and costs were discounted to 2023, with a rate of 3% [17].

**Fig 2:**
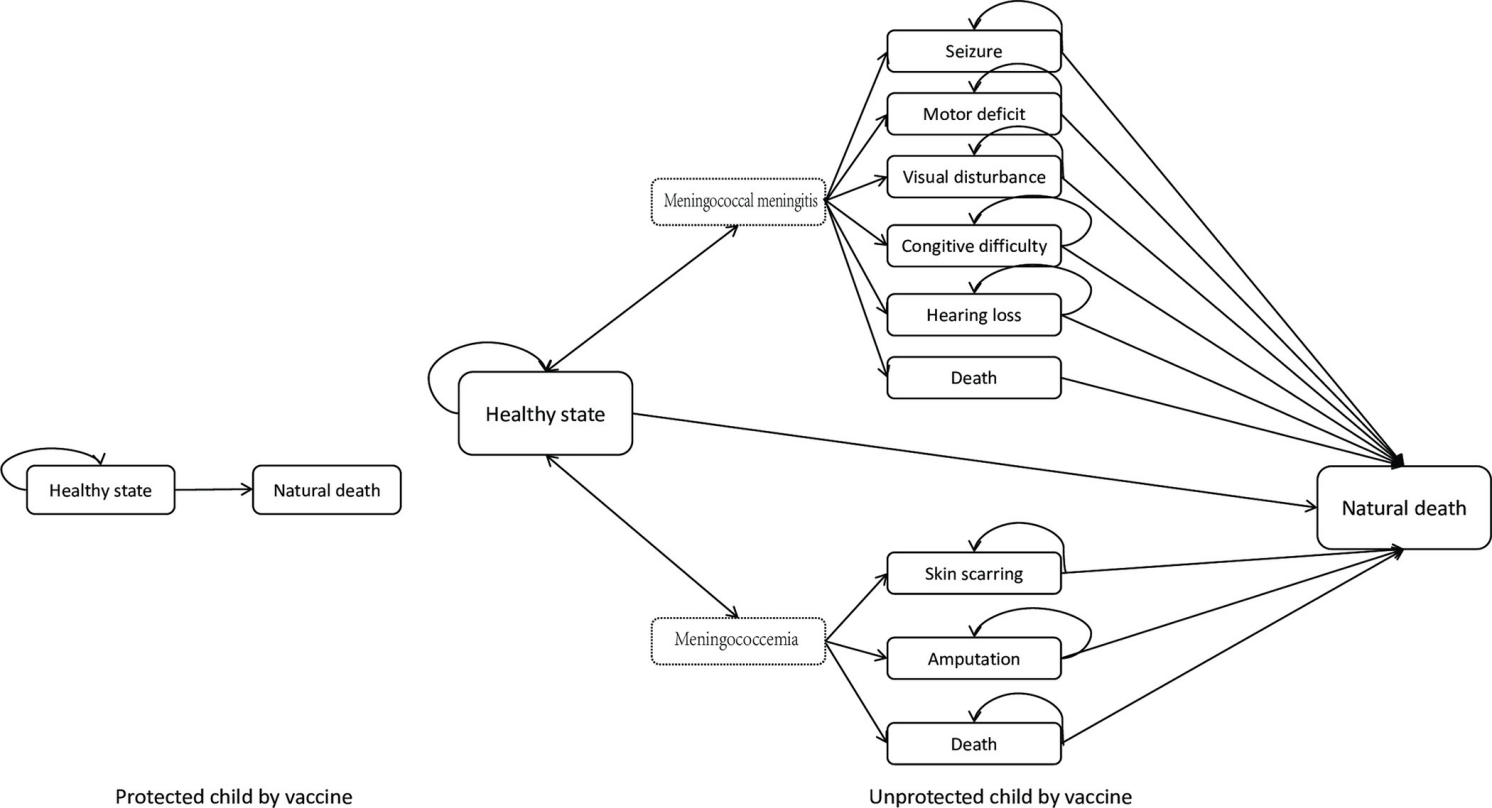
Markov chains simulating the health status of individuals.

The structures of the model and Markov chains were presented in Fig 2. The states in Markov chain comprised eleven states for the unprotected population, with “meningococcal meningitis” and “meningococcemia” serving as intermediate stages for better understanding of the transition. The unprotected individuals were firstly in a healthy/susceptible state. All meningococcal disease case were assumed to be treated in hospital and the prognosis included recover to the healthy state, survive with sequelae and death for meningococcal disease.

### 2.3 Parameters

The parameters and data source were presented in Table 1. The mortality for all natural causes was derived from statistics yearbook of Zhejiang province. The proportion of serogroups observed in all meningococcal disease was derived from a study by China CDC [6]. The incidence rates of meningococcal meningitis was obtained from the report on China notifiable infectious diseases of 2021 [18]. The probabilities of sequelae for meningococcal meningitis were derived from a meta analysis and the fatality rate of meningococcal meningitis was collected from a systematic review [19, 20]. Since the incidence and case fatality of meningococcemia were limited home and abroad, we used the data from an Asian country similar with China [21]. The probabilities of sequelae for meningococcemia were obtained from two studies [22, 23].

**Table 1:** Parameters used in the model.

Variables	Base value	Range	Distribution	Source
**EPIDEMIOLOGY**				
***Incidence (/100000)***				
Meningococcal meningitis	1.90	0.88–3.76	Beta	15
Meningococcemia	0.80	0.37–1.59	Beta	18
***Serogroup distribution(%)***				
MenA	4.73	Base ± 20%	Beta	5
MenC	22.97	Base ± 20%	Beta	5
MenY	1.69	Base ± 20%	Beta	5
MenW	6.08	Base ± 20%	Beta	5
***Proportion of sequelae in meningitis(%)***				
Hearing loss	4.60	3.0–7.3	Beta	16
Cognitive difficulties	2.90	1.0–7.2	Beta	16
Visual disturbance	2.70	1.1–4.1	Beta	16
Motor deficit	1.80	1.1–4.1	Beta	16
Seizure	0.90	0.1–2.0	Beta	16
***Proportion of sequelae in meningococcemia(%)***				
Skin scarring	9.00	Base ± 20%	Beta	19–20
Amputations	28.60	Base ± 20%	Beta	19–20
***Case fatality(%)***				
Meningococcal meningitis	4.80	2.86–7.31	Beta	17
Meningococcemia	26.30	13.20–40.00	Beta	18
***All-cause mortality(‰)***				
<1 yrs	2.80	Base ± 20%	Beta	Health Yearbook
1–4 yrs	1.20	Base ± 20%	Beta	Health Yearbook
5–9 yrs	0.14	Base ± 20%	Beta	Health Yearbook
***Vaccine wastage rate***	0.05	Base ± 20%	Beta	21
***Vaccination coverage***	0.9	Base ± 20%	Beta	13
**COST ($)**				
***Vaccination program***				
MPV-A price/dose	0.21	Base ± 20%	Gamma	-
MPV-AC price/dose	1.73	Base ± 20%	Gamma	-
MPV-ACYW price/dose	16.82	Base ± 20%	Gamma	-
MCV-ACYW price/dose	60.883	Base ± 20%	Gamma	-
Syringe price/dose	0.17	Base ± 20%	Gamma	-
Service cost/dose	3.72	Base ± 20%	Gamma	22
***Economic burden of the disease***				
Meningococcal meningitis	8373.31	Base ± 20%	Gamma	23
Meningococcemia	11866.44	Base ± 20%	Gamma	24
Hearing loss	22624.28	Base ± 20%	Gamma	CHIRA
Cognitive difficulties	36544.50	Base ± 20%	Gamma	CHIRA
Visual disturbance	6435.34	Base ± 20%	Gamma	25
Motor deficit	1755.34	Base ± 20%	Gamma	CHIRA
Seizure	832.91	Base ± 20%	Gamma	CHIRA
Amputations	4659.92	Base ± 20%	Gamma	26
Productivity lost due to death	111626.70	Base ± 20%	Gamma	6th Census of China
Discount rate	3	2.25–5.00	Uniform	14
**UTILITY**				
***Health***	1	-	-	-
***Death***	0	-	-	-
***Meningococcal meningitis***	0.97	0.59–1.00	Beta	29
***Meningococcemia***	0.99	0.78–1.00	Beta	29
***Sequelae***				
Hearing loss	0.91	0.83–1.00	Beta	30
Cognitive difficulties	0.62	0.51–0.73	Beta	30
Visual disturbance	0.26	0.19–0.33	Beta	31
Motor deficit	0.67	0.55–0.79	Beta	30
Seizure	0.83	0.75–0.91	Beta	30
Skin scarring	1	Base ± 20%	Beta	32
Amputations	0.69	0.69–0.8	Beta	33
**VACCINE EFFICACY (%)**				
***MPV-A***				
MenA	55.56	Base ± 20%	Beta	27
***MPV-AC***				
MenA	75.36	Base ± 20%	Beta	28
MenC	94.20	Base ± 20%	Beta	28
***MPV-ACYW***				
MenA	82.15	Base ± 20%	Beta	27
MenC	90.57	Base ± 20%	Beta	27
MenY	50.84	Base ± 20%	Beta	27
MenW	54.88	Base ± 20%	Beta	27
***MCV-ACYW***				
MenA	91.42	Base ± 20%	Beta	27
MenC	88.76	Base ± 20%	Beta	27
MenY	88.17	Base ± 20%	Beta	27
MenW	99.41	Base ± 20%	Beta	27

The cost in this model comprised both vaccination program and economic burden of meningococcal disease. The cost of vaccination program comprised vaccine and syringe procurement, vaccine services. Prices for MPV-A and MPV-AC were $0.209 per dose and $1.728 per dose, according to procurement price by China CDC. Prices for MCV-ACWY and MPV-ACWY were $60.883 per dose and $16.815 per dose, according to the purchase price as the voluntary vaccine. The price of syringe was $0.174 per dose, followed data from centralized procurement by China CDC. The wastage rate for vaccines was set to be 5% [24]. The cost of vaccine services was 3.714 per dose, including vaccine storage and transportation, staff training, personnel wage, and cold chain management, was derived from a survey study conducted by the China CDC [25]. The economic burden of meningococcal disease included the medical treating, costs arising from sequelae, and loss of productivity due to sequelae or death. Cost of medical treating for meningococcal meningitis was derived from a survey conducted in China [26]. Cost of medical treating for meningococcemia was estimated from a review providing the cost for meningitis and meningococcemia in US [23]. Costs arising from sequelae of meningococcal disease, such as the treatment and special education expenses for cognitive difficulty, cochlear implants and maintenance for hearing loss, special education expenses for visual disturbance, were calculated using a discounted annual additional cost for individuals up to the life expectancy, with referring the Chinese health insurance research association (CHIRA) or relevant surveys implemented in China [27, 28]. The loss of lifetime productivity due to disability or death was estimated using data from the 6th Census of China.

The vaccine efficacy of MPV-A, MPV-ACWY, MCV-ACWY for every serogroup was collected from a phase III clinical trial of MCV-ACWY conducted in China [29]. The vaccine efficacy of MPV-AC for serogroup A and serogroup C was derived from another Chinese report [30]. Individuals protected by vaccines referred to those who were vaccinated and had produced a protective effect, while individuals not protected by vaccines include those who have not been vaccinated and those for whom vaccination has not produced a protective effect.

The utility value of 1 was assigned to health status, while a value of 0 was assigned to death. The utilities for meningitis, meningococcemia and the relevant sequelae were obtained from the existing utility studies on bacteremia, sequelae after meningitis and blindness [31–35].

### 2.4 Sensitivity analysis

The one-way sensitivity analysis was used in this study to determine the uncertainties in input variables and verify the robustness of the model. The range of values for the parameters were presented in Table 1. If the range of values for parameters was not available, a deviation of ± 20% was adopted. A probabilistic sensitivity analysis (PSA) was implemented through assigning distributions to key parameters and performing Monte Carlo simulations with 10000 iterations. The effects of variations in specific parameter on the model were depicted using tornado diagrams. The results of PSA were presented in the form of the cost-effectiveness acceptability curve. Moreover, the study explored the precise prices of voluntary vaccines that would make the alternative meningococcal vaccination strategy be cost-effective. We also compared the cost-effectiveness of the meningococcal vaccination strategies to the willingness-to-pay (WTP) threshold of one time of the GDP per capita of Zhejiang province in 2023 ($17318.98).

### 2.5 Ethics approval and consent to participate

This study was exempted for ethics approval as it did not contain any personal information and include no human research participants.

## 3. Results

The health impacts and costs of the current and alternative meningococcal vaccination strategies were presented in Table 2. For the target cohort from birth to 9 years of age, there would be 3092 meningococcal disease cases, 403 sequelae and 351 deaths under the current vaccination strategy. In comparison, the alternative strategy indicated the potential to reduce the meningococcal disease burden, with a reduction of 513 cases, 53 sequelae and 47 deaths. Replacing meningococcal vaccination strategy with the alternative strategy could result in an additional 5056.61625 QALYs and 85455851.46 $ for the target cohort, with an ICER of $16899.81 /QALY. The alternative strategy was deemed cost-effective as the calculated ICER under the threshold of one time of the GDP per capita of Zhejiang province in 2023($17318.98).

**Table 2:** Incremental cost and effectiveness of alternative meningococcal vaccination strategy compare to current practice.

	MPV-A and MPV-AC	MCV-ACWY and MPV-ACWY
Meningococcal disease(number of case)	3092	2579
Sequelae(number of case)	403	350
Death(number of case)	351	304
Individual effectiveness(QALYs)	29.95418	29.967305
Incremental effectiveness for target cohort(QALYs)	-	5056.62
Individual cost($)	12.06	233.87
Incremental cost for target cohort($)	-	85455851.46
ICER($/QALY)	-	16899.81

**Fig 3:**
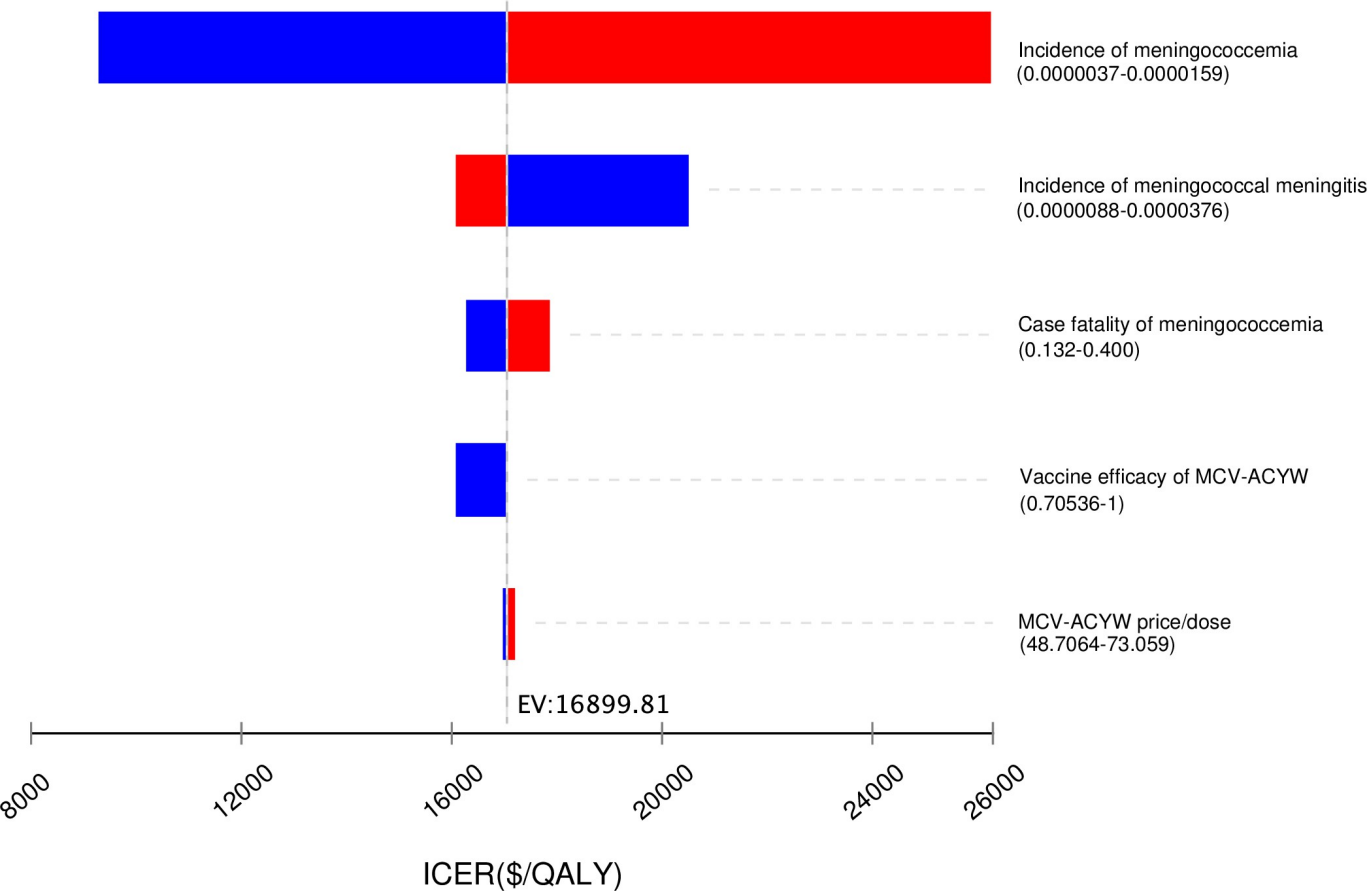
Tornado diagrams for replacing the current meningococcal vaccination strategy with the MCV-ACWY and MPV-ACWY strategy.

**Fig 4:**
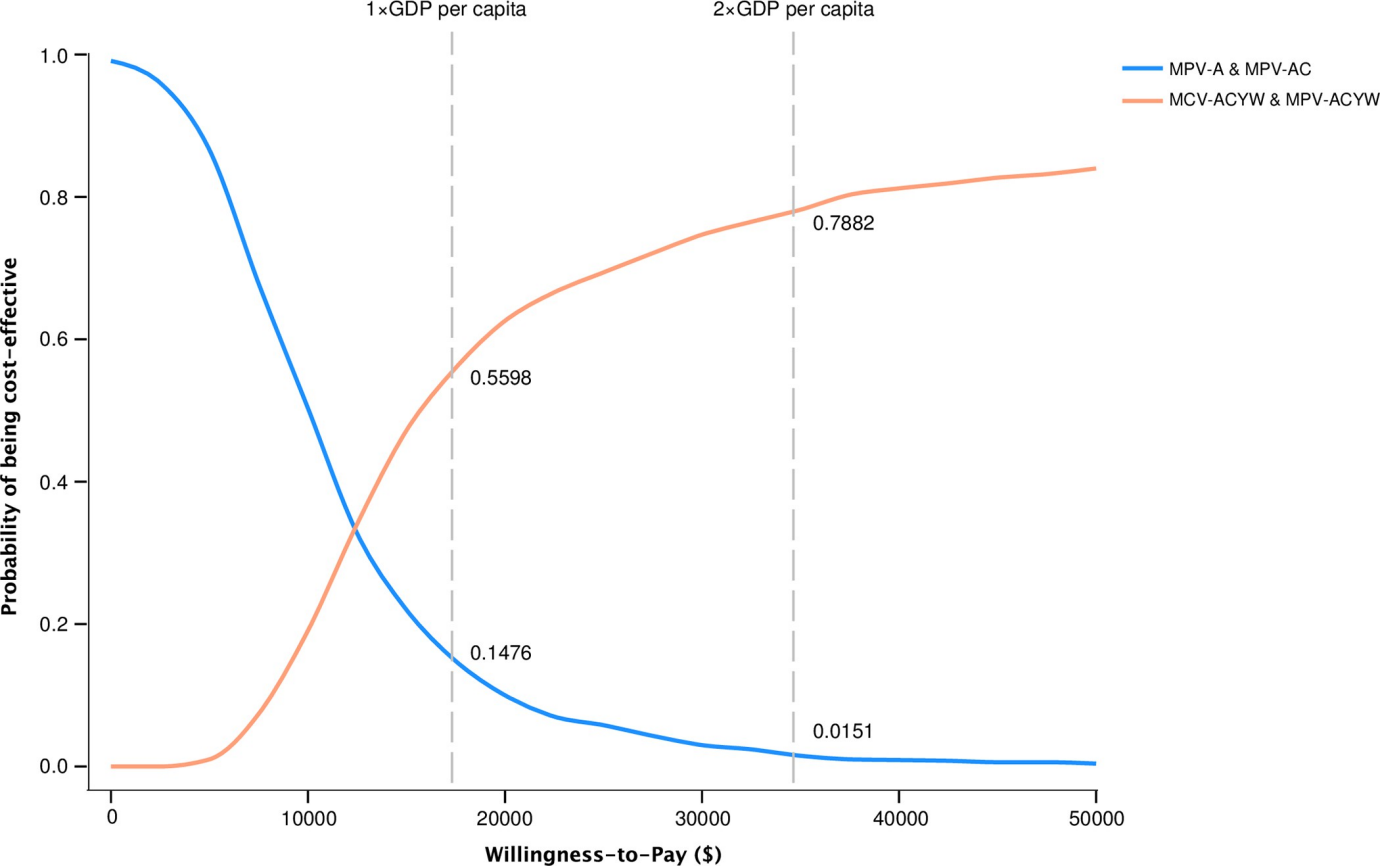
Cost-effectiveness acceptability curves for the current meningococcal vaccination strategy, MCV-ACWY and MPV-ACWY strategy.

The main factors impacted the cost-effectiveness included the incidence of meningococcemia, the incidence of meningococcal meningitis, the case fatality of meningococcemia, the vaccine efficacy of MCV-ACWY and the price of MCV-ACWY per dose (Fig 3). Through the PSA analysis, the alternative strategy had a higher probability of being cost-effective. At the threshold of one time of the GDP per capita, the estimated probability of cost-effectiveness was 14.76% for the current strategy and 55.98% for the alternative strategy. At the threshold of two times of the GDP per capita, the estimated probability of cost-effectiveness was 1.51% for the current strategy and 78.82% for the alternative strategy (Fig 4).

## 4. Discussion

Considering the incidence of meningococcal disease and the social-economic development in different provinces varying, meningococcal vaccination strategy could be designed based on the provincial setting to implement appropriate strategy at provincial level. This analysis was firstly to evaluate the cost-effectiveness of replacing the current meningococcal vaccination strategy with an alternative strategy of using non-NIP vaccines at provincial level in China. Our findings demonstrated that the alternative strategy using 3-dose of MCV-ACWY and 2-dose of MPV-ACWY in children provided the higher health benefits over the current practice. The cost of the alternative strategy rose with health benefits, but it was still cost-effective compared to the current practice. Furthermore, variations in the parameters did not affect the ICER for the alternative strategy replacement through the sensitivity analysis, indicating the robustness of model.

Prior studies on the cost-effectiveness of meningococcal vaccination strategies had limited comparability to this study, as MPV-A, MPV-AC, MPV-ACWY were rarely used in other countries at present. Moreover, the diverse study time, population, vaccination schedule, epidemiology of meningococcal disease and social-economic development in the study areas failed the comparison of the findings between the previous and the present analysis. A cost-benefit analysis against MPV-4 was implemented in college students in the US in 1995, concluding that strategy was not desirable [33]. Another report from West Africa assessed the mass vaccination with MPV-AC, concluding it was cost-effective [34]. There were also several reports on evaluating the economics of MCV-ACWY [35–37]. In Canada, three studies had compared the use of MCV-ACWY to that of polysaccharide conjugate vaccine against MenC (MCV-C) [38–40]. Of these studies, two revealed that the replacement with MCV-ACWY was not cost-effective as the strategy with MCV-ACWY yielded the higher cost per QALY gained. The other one observed lower ICERs for the MCV-ACWY strategy in adolescents than in infants, and the strategy of vaccinating MCV-C in infants and MCV-ACWY in adolescents was even less cos-effective than vaccinating both target groups with MCV-C. It indicated that the cost-effective analysis of meningococcal vaccination strategy should be implemented for different age groups, supporting the decision-making for expanding the vaccination program to adolescents for more universal protection against meningococcal disease in future. Anther cost-effectiveness study on meningococcal vaccination compared the alternative strategies with the current strategy in China [41]. It indicated that the ICER was approximately $1.5 million/QALY when using alternative strategy included 3 doses of MCV-ACWY at 3,4,5 months of age and 1 dose of MPV-ACWY at 6 years of age. However, this alternative was not cost-effective as it exceeded the threshold of cost-effective. There might be two reasons for the difference between this study and our analysis. First is the vaccination schedule was not same as there were two doses of MPV-ACWY in our model and it would improve the effectiveness and protective duration for vaccinees. Second, the threshold of cost-effective was set as three times of the GDP per capita in China in 2019 ($30,475) in the previous report but was not same as this analysis($17318.98).

Study conducted in the Norway assessed the inclusion of MCV-ACWY into NIP in adolescents, drawing a conclusion of being cost-effective under a high WTP threshold [42]. The Norwegian study adopted a threshold of €86000/QALY, which was based on the local GDP per capita in 2021 and was much higher than the threshold in our analysis. A 50% discount for the price of MCV-ACWY included in the NIP was assumed in the study from Norway, reducing the ICER and contributing to the cost-effectiveness. Given the bargaining power of the large-scale procurement, the price of voluntary vaccines would decrease after their inclusion into NIP, leading to a lower ICER and probable cost-effectiveness. In this study, the formulation of WTP threshold would indicate the local benchmark for economic evaluation, incorporating the present social- economic development and the importance of individual’s health under the reference to WHO guidance [17]. Another study evaluated the MCV-ACWY as an alternative to MCV-A in 26 African countries in the meningitis belt [43]. It indicated that the MCV-ACWY vaccination program was cost-effective in 14 countries if the incidence of meningococcal disease was around 50/100000 person-year, while was cost-effective in all 26 countries if the incidence was at 150/100000 person-year. More cases could be averted by using non-NIP vaccines like MCV-ACWY when the incidence of meningococcal disease was higher. The increased cost due to the expensive voluntary vaccines included into NIP could be offset more by the decreasing disease burden caused by averting cases and the complications, allowing for reducing the incremental cost and achieving the lower ICER in final. On the contrary, the low incidence of meningococcal disease used in this model could increase the ICER in our results. Passive surveillance from Chinese national notifiable disease reporting system might suffer from under-reporting. Meningococcal disease would be misdiagnosed or underreported as its initial symptoms were atypical [2]. Cost-effectiveness using underestimated incidence might underestimated the benefits of economic and health from vaccination, resulting in a higher ICER.

The current meningococcal vaccination strategy in NIP using MPV-A and MPV-AC had achieved the control goal with a low cost, reducing the incidence of meningococcal disease and maintaining it. However, meningococcal disease could cause devastating cost for family in medical treatment and productivity loss for society. The capacity of current practice needed to be exceeded for further reducing the incidence. Neisseria meningitidis serogroups other than MenA and MenC could not be effectively prevented by current meningococcal vaccines in NIP, leaving a risk of meningococcal disease caused by the other serogroups. The findings of our analysis demonstrated that the alternative strategy could reduce the incidence of meningococcal disease and was deemed cost-effective. We assumed that the alternative strategy would be more cost-effective since the vaccine price might be discounted for inclusion of vaccine in the NIP. In recent years, MenB became the predominant prevalent serogroup and was responsible for 52.4% of meningococcal disease cases in China [4]. Currently, No vaccine against MenB was available in mainland China [8]. Otherwise, over half of the meningococcal disease cases will be averted if we include MenB vaccine into NIP. However, MenB vaccine was licensed in 58 countries, of which 15 included MenB vaccine targeting infants in the NIP [44]. A cost-effectiveness analysis indicated the value of MenB vaccination among infant, verifying it was cost-effective at a threshold of £20,000/QALY [45]. The introduction of MenB vaccine into China might result in significant decrease in the number of meningococcal disease and relieve the relevant economic burden. We recommend that future cost-effectiveness analysis should be implemented to evaluate the health and economic influence on approving MenB vaccine in China as a NIP vaccine.

There were several limitations for this study. First, some of the parameters were from other countries as local data were not available. It would not accurately reflect the true local conditions. We recommend that further economic evaluations should be conducted when the relevant information become richer. Second, the waning of vaccine efficacy was not considered. Studies on the waning of efficacy for meningococcal vaccines were limited and there was no relevant report from China. We assumed that the duration of protection could last till the next vaccine dose and the waning of efficacy would not affect the results. Third, the probability of switching among different states did not vary over time or with the number of cases, therefor the effect of herd immunity could not be estimated. Since the unvaccinated individuals might be indirectly protected from meningococcal disease due to the vaccinated individuals, the number of cases [46], sequela and death averted by meningococcal vaccination in this study might be underestimate.

## 5. Conclusions

The current meningococcal vaccination strategy in Chinese NIP was proved to be effective in reducing the incidence of meningococcal disease. The alternative vaccination strategy using MCV-ACWY and MPV-ACWY could increase the health benefits with a cost-effective manner. We recommend that the Zhejiang provincial government consider the inclusion of MCV-ACWY and MPV-ACWY in the NIP to enhance coverage and protection against diverse serogroups and reduce economic burden due to the disease.
