# Correction: Economic evaluation on meningococcal vaccination strategies among children under nine years of age in Zhejiang province, China

**DOI:** 10.1371/journal.pone.0320386

**Published:** 2025-03-10

**Authors:** Jianyong Shen, Chai Ji, Xiaofu Luo, Yu Hu

There are errors in the author affiliations. The correct affiliations are as follows:

Jianyong Shen^1^, Chai Ji^2^, Xiaofu Luo^1^, Yu Hu^3^

**1** Institute of Immunization and Prevention, Huzhou Municipal Center for Disease Control and Prevention, Huzhou, China, **2** Department of Children Healthcare, Children’s Hospital Affiliated to Zhejiang University School of Medicine, Hangzhou, China, **3** Institute of Immunization and Prevention, Zhejiang Provincial Center for Disease Control and Prevention, Hangzhou, China.

## References

[1] Shen J, Ji C, Luo X, Hu Y. Economic evaluation on meningococcal vaccination strategies among children under nine years of age in Zhejiang province, China. PLoS One. 2024;19(9):e0310274. doi: 10.1371/journal.pone.0310274 39250492 PMC11383224

